# Fabry disease: a rare disorder calling for personalized medicine

**DOI:** 10.1007/s11255-024-04042-4

**Published:** 2024-04-13

**Authors:** Sarah Lerario, Luigi Monti, Irene Ambrosetti, Agnese Luglio, Andrea Pietra, Valeria Aiello, Francesca Montanari, Antonio Bellasi, Gianluigi Zaza, Antonio Galante, Davide Salera, Irene Capelli, Gaetano La Manna, Michele Provenzano

**Affiliations:** 1 Nephrology, Dialysis, and Kidney Transplant Unit, IRCCS Azienda Ospedaliero-Universitaria Di Bologna, Bologna, Italy; 2 Medical Genetics Unit, IRCCS Azienda Ospedaliero-Universitaria Di Bologna, Bologna, Italy; 3 Servizio Di Nefrologia, Ospedale Regionale Di Lugano, Ente Ospedaliero Cantonale, Ospedale CivicoVia Tesserete 46, 6903 Lugano, Switzerland; 4 Università Della Svizzera Italiana (USI), Lugano, Switzerland; 5 Department of Pharmacy, Health and Nutritional Sciences, University of Calabria, 87036 Rende, CS Italy

**Keywords:** FD, Treatment, Enzyme replacement therapy, Chaperone therapy, Gene therapy, mRNA therapy

## Abstract

Fabry Disease (FD) is a genetic disease caused by a deficiency in the activity of lysosomal galactosidase A (α-GalA), an enzyme responsible for the catabolism of globotriaosylceramide (Gb3). Since lysosomes are present throughout the body and play a crucial role in catabolism and recycling of cytosolic compounds, FD can affect multiple organs and result in various symptoms, including renal, cardiovascular, neurological, cutaneous, and ophthalmic manifestations. Due to the nonspecific symptoms and the rarity of FD, it is often diagnosed late in life. However, introducing targeted therapies such as enzyme replacement therapy (ERT) and chaperone therapy has significantly improved FD's natural history and prognosis by restoring α-GalA enzyme activity. Despite the advancements, there are limitations to the currently available therapies, which has prompted research into new potential treatments for FD, including alternative forms of enzyme replacement therapy, substrate reduction therapy, mRNA therapy, and genetic therapy. In this review, we analyze the epidemiology, pathophysiology, and treatment of FD, with particular emphasis on promising therapeutic opportunities that could shift the treatment of this rare disease from a standardized to a personalized approach soon.

## Introduction

Fabry Disease (FD) is a rare X-linked genetic disease characterized by the reduced activity of lysosomal galactosidase A (α-GalA), a key enzyme involved in globotriaosylceramide (Gb3) catabolism.

The ubiquitous presence of lysosomes, central to the breakdown and recycling of cellular materials, means that FD's impact is widespread, affecting numerous organs and presenting a spectrum of phenotypes shaped by the extent of renal, cardiovascular, neurological, skin, and eye involvement[1].

Clinically, FD manifests primarily in two forms: the early-onset “classic form” and the attenuated (so-called atypical or late) form. The classic variant is marked by negligible or low (< 1%) α-GalA activity, leading to early and extensive multi-organ complications. In contrast, the attenuated form emerges later in life with variable manifestations contingent on the remaining α-GalA activity levels. Notably, female carriers might experience a disease burden comparable to males or may remain symptom-free, depending on the nature of the GLA mutation and on the X chromosome inactivation (lyonization) that increases phenotypic variability [2].

In recent years, significant advances have been observed in the comprehension of FD, from pathogenesis to natural history, which has led to increased awareness and new diagnoses.

Early detection of FD is essential, considering the progressive accumulation of Gb3 in the target organs. It allows for early disease staging and prompts tailored therapy to avoid organ damage.

For example, a multidisciplinary team, which may include nephrologists, cardiologists, neurologists, ophthalmologists, and geneticists, is required for patient evaluation and assessment of disease progression risk. Prognostic scores like FASTEX, MSSI, and DS3 [1–3], combining clinical, radiological, and laboratory information, are available for patients’ follow-up over time and to evaluate the stability or progression of the pathology [4]. The main therapeutic aims of FD treatments are the amelioration of symptoms and the prevention of Gb3 deposition in the target organs to reduce disease progression and increase life expectancy. Indeed, ERT replaces the α-GalA deficiency and reduces Gb3 accumulation, while migalastat, the chaperone therapy available on the market, corrects the misfolded α-GalA. Both treatments have been proven to alleviate patients’ outcomes and survival [5].

Regrettably, the approved therapies for FD have some limitations; for example, ERT has limited tissue penetration and cannot pass the blood–brain barrier. Moreover, it can induce adverse infusion reactions, and its efficacy could be limited due to the development of anti-drug-neutralizing antibodies. On the other hand, the chaperon therapy molecule binds only to specific α-GalA domain, so it is a therapeutic option only for patients with amenable GLA mutations, and it is not recommended in pregnancy and in subjects with a reduced glomerular filtration rate (eGFR) less than 30 ml/min/1.73m^2^[5].

The quest for more personalized and productive treatments has sparked research into novel therapeutic avenues, such as innovative ERT formulations, substrate reduction therapy, mRNA therapy, and gene therapy, heralding a new epoch of individualized medicine for FD [9]. In this review, we analyze the epidemiology, pathophysiology, and therapies of FD, explicitly focusing on promising therapeutic possibilities that could move the treatment of this rare disease from a standardized to a personalized treatment.

## Methods

The authors conducted a comprehensive bibliographic search using Pubmed, Scopus, and Google Scholar databases. Specific keywords were used to explore Fabry Disease, its pathogenesis, symptoms, genetics, and treatment. The search was extended to synonyms and matching terms. Only original articles and reviews written in English and published in peer-reviewed journals were selected.

## Epidemiology

The overall prevalence of the classic form of FD is estimated at around 1 in 40,000 to 170,000 births [6]. However, the actual prevalence of this condition is likely higher due to the challenges in diagnosing a disease with a wide range of clinical presentations and ages at presentation, particularly the atypical or attenuated form.

Newborn screening programs for FD have been developed to make an early diagnosis of the disease. However, as Gragnaniello et al. [7] recently summarized in a literature review, newborn screening is controversial. Among the advantages of such screening is the opportunity to obtain an early diagnosis of a treatable disease, expand screening, counseling, and, eventually, therapy for other family members. Of course, there are some disadvantages, including the possibility of detecting Variants of Uncertain Significance (VUS) and benign variants, the early diagnosis of late-onset variants, which may have a negative psychological impact on patients and parents, and the lack of well-defined guidelines for follow-up and for the timing of therapy initiation. Among the disadvantages, it is essential to mention the difficulty in identifying heterozygous females through enzymatic assays.

Between 2003 and 2005, in Northwest Italy’s Piemonte region, a pilot Newborn Screening (NBS) project for lysosomal storage diseases focused on Fabry disease (FD) was started. They screened 37,104 male newborns using a fluorometric assay. Among them, twelve neonates tested positive for a specific GLA variant (occurring in 1 out of 3,092 males). Interestingly, only one patient carried a variant linked to the classic phenotype, highlighting a notable prevalence of later-onset forms [8]. Another Italian study recently analyzed dried blood spots (DBS) from 173.342 newborns and found an estimated incidence of FD ranging from 1:1.145 to 1:18.436 [9].

An interesting epidemiological study was recently performed on 200,643 samples from the UK Biobank. Exome sequencing data were collected to determine the overall prevalence of GLA variants. The results showed that the prevalence of the FD-causing variant was 1 in 5,573, which aligns with the studies described above [10].

There are challenges in establishing a definitive and unambiguous prevalence of FD. In the non-classic forms of FD, symptoms can be very mild, presenting only later in life, and highly unspecific (e.g., stroke, left ventricular hypertrophy, chronic renal disease…). Molecular analyses are not always of help since there are more than 1.000 different reported variants in the GLA gene, and, in many cases, genetic testing identifies very rare or private variants of unknown significance. Furthermore, testing for levels of enzymatic activity often lacks sensitivity, especially in females, and borderline results can be challenging to interpret. Establishing the diagnosis in women, who usually present a milder and unspecific phenotype, is particularly difficult. These difficulties can lead to missed diagnoses and an underestimation of FD's prevalence.

## Pathophysiology

**Fig. 1:**
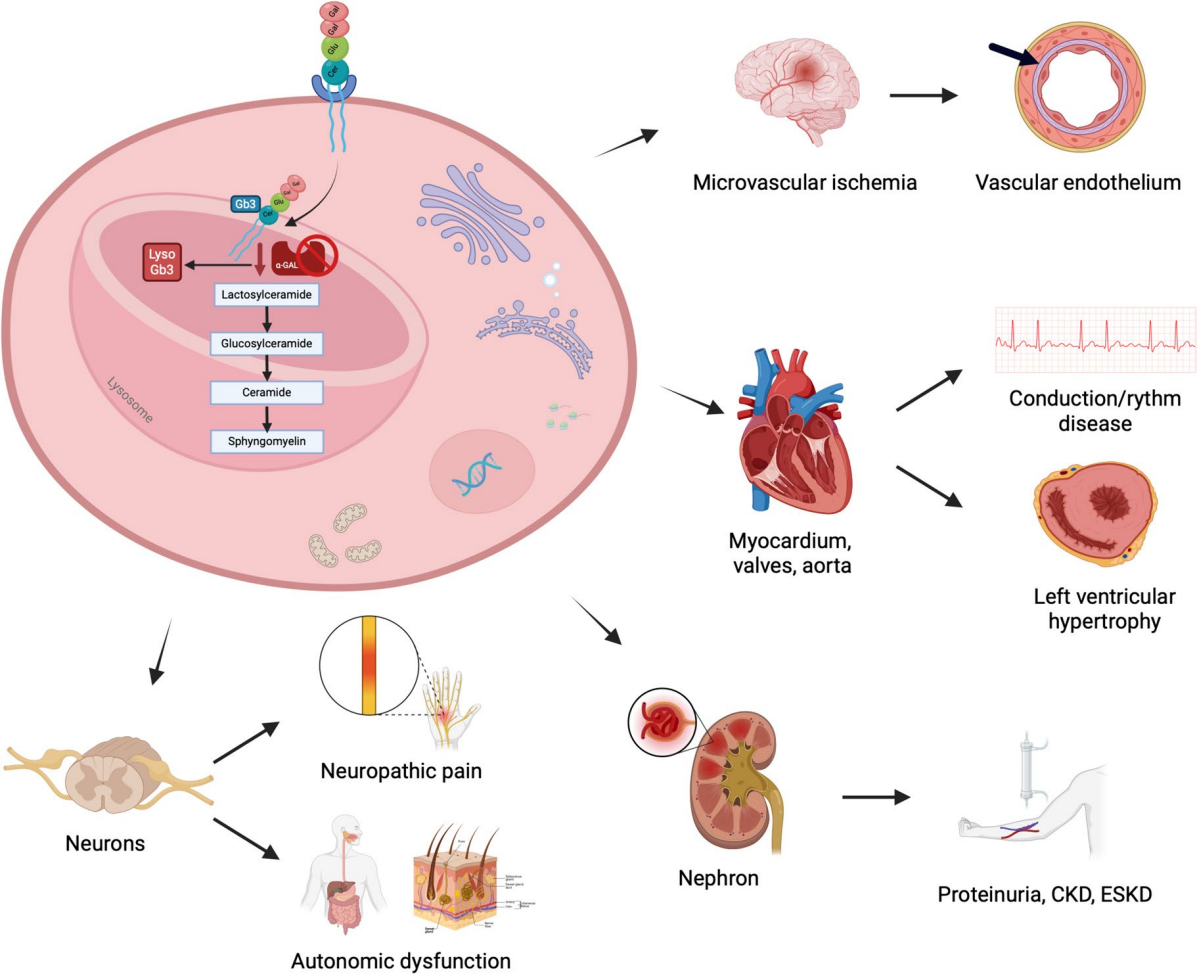
Pathophysiology of Fabry disease. Lysosomal accumulation of glycosphingolipids in different cell lines is responsible for organ damage in Fabry disease

Mutations in the GLA gene are responsible for the absence or reduced activity of the enzyme α-GalA, which consequently alters the metabolism of some glycosphingolipids and accumulates metabolites in many tissues, such as globotriaosylceramide (Gb3) and its deacylated hydrophilic derivatives, such as lysoGb3 (or globotriaosylsphingosine) and its analogs (Fig. 1) [4].

The occurrence of clinically significant FD is associated with α-GalA activity falling below a threshold level of approximately 30–35% of the average expected value [11]. Males with the classic form exhibit a residual enzyme activity of less than 1% of the normal value, whereas females and those with the attenuated forms show higher values. The likelihood of developing complications related to the disease is primarily determined by enzyme activity [12, 13]. It is known that α-GalA deficiency causes the accumulation of Gb3 in lysosomes in various tissues, but the mechanism(s) by which this occurs remains unclear [12]. Especially, Gb3 accumulation is increased in vascular endothelium, vascular smooth muscle cells, and pericytes, potentially leading to several health issues, such as cell death, vascular occlusion, ischemia, and infarction [11]. Additionally, elevated levels of Gb3 are also found in the autonomic ganglia, dorsal root ganglia, renal glomerulus, renal tubule and interstitial cells, cardiac muscle cells, corneal endothelial cells, valvular fibrocytes, cardiac conduction cells, resulting in a range of disease symptoms. However, the disease is not solely attributed to the accumulation of Gb3, and other factors, though not fully understood, may play a role in the overall clinical picture. The accumulation of Gb3 not only leads to mechanical effects but also triggers inflammation. When α-GalA is deficient, the degradation of lipid antigens is limited, allowing for their accumulation with the activation of natural killer T cells that contribute to chronic inflammation and autoimmunity [14]. Additionally, the accumulation of Gb3 can impair endocytosis and autophagy (crucial recycling pathways that promote cell survival), induce apoptosis, and disrupt mitochondrial energy production [15]. Another characteristic of FD is the significant increase in water-soluble deacylated Gb3, also referred to as globotriaosylsphingosine (lyso-Gb3), and its analogs: they are believed to possess cytotoxic, proinflammatory, and profibrotic properties, further contributing to the development of the disease [16]. In particular, lysoGb3, increased in both the blood and urine of affected patients, is responsible for smooth muscle cell proliferation, which leads to increased thickness and arterial stiffness. Additionally, it can induce pain in the extremities by damaging nociceptive neurons. At the same time, it may cause podocyte loss and glomerular fibrosis in the kidney and inhibit endothelial nitric oxide synthase (eNOS), promoting vasculopathy [17, 18]. Furthermore, the dosage of lyso-gb3 is helpful in clinical practice: it correlates with the severity of the disease, allows confirmation of the diagnosis in case of detection of a genetic variant of uncertain significance, and evaluates the response to therapy [13].

## Cardiorenal and nervous system involvement in FD

**Fig. 2:**
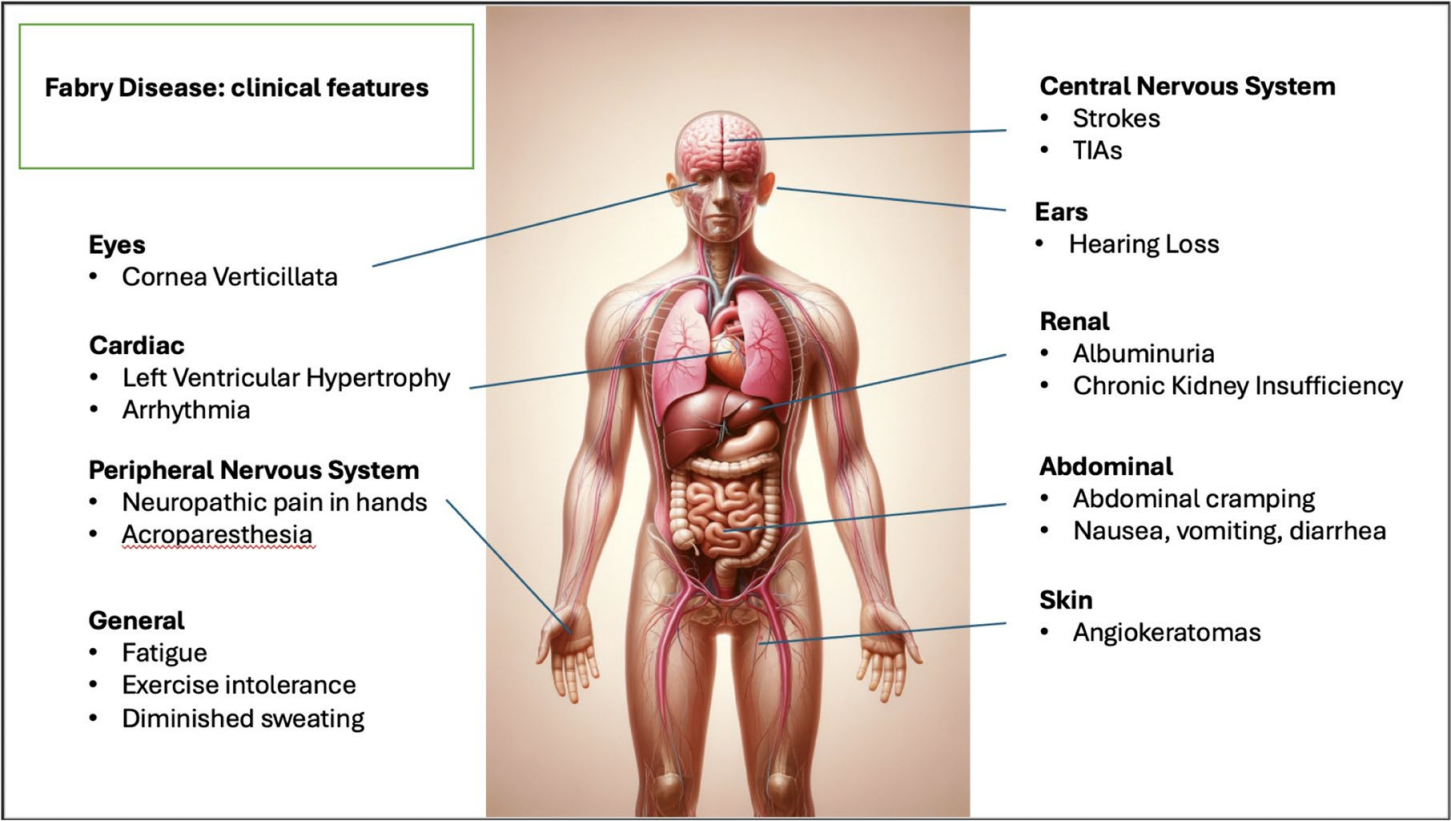
Clinical features of Fabry disease

Clinical features in FD are depicted in Fig. 2. The significant morbidity and mortality rate in FD can be attributed to the chronic and progressive damage to the kidneys, heart, and central nervous system.

Renal involvement has been identified through vacuolated epithelial cells and podocytes in both the glomerulus and distal tubules. The light microscope shows lipid inclusions visible as clusters of intracellular vacuoles. Intracytoplasmic irregular lamellar bodies containing Gb3, called “zebra bodies,” are visible through electron microscopy. Over time, the glomeruli become increasingly sclerotic, and mesangial widening, tubular atrophy, and interstitial fibrosis can be observed in kidney biopsies. Notably, proteinuria often precedes damage to the renal system as it can occur as early as adolescence, further promoting renal damage and end-stage kidney disease (ESKD) in the fourth decade of life.

Oxidative stress, inflammation, and endothelial dysfunction are other kidney, brain, and cardiovascular damage factors. Increased circulating levels of ROS (reactive oxygen species), interleukin-1, and decreased availability of NO (nitric oxide) promote arterial intimal hyperplasia as well as deposition of abnormal extracellular matrix and hyaline material in the context of the arterial wall, resulting in vascular calcification, arterial stiffness, and elevated differential pressure [19, 20]. Gb3 and lyso-Gb3 accumulation also contribute to the epithelial-mesenchymal transition (EMT) through the production of TGF-beta (transforming growth factor-beta). Hence, epithelial tubular cells in the kidneys modify their phenotype into myofibroblasts and synthesize extracellular matrix, contributing to fibrotic damage and organ failure [21].

An elegant work by Tondel et al. [22] showed that Gb3 and lyso-Gb3 accumulation and fibrotic damages occur in the kidneys before the occurrence of microalbuminuria or proteinuria. Indeed, in a series of 8 kidney biopsies of children (aged 4 to 16 years) with FD, authors found Gb3 accumulation in several cellular types with a particular podocyte involvement, which undergo a segmental effacement. In this series, participants exhibited normal renal function and no signs of albuminuria or proteinuria [22]. This could have implications on the controversial issue about the timing of treatment starting in young male patients or females considered “asymptomatic.”

Accumulation of Gb3 and lyso-Gb3 also occurs in the heart and affects all cardiac cell types and tissues such as the myocytes, the vascular endothelial cells, smooth muscle cells, the endocardium, fibroblasts of valvular apparatus, and the conduction system [14]. The typical cardiomyopathy of FD is primarily characterized by left ventricular hypertrophy with restrictive cardiomyopathy features. In almost half of the affected patients, valvular abnormalities have been documented, although they are seldom classified as severe. Upon ultrastructural investigation, it was discovered that cardiomyocytes were larger and contained vacuoles filled with Gb3 in their perinuclear regions. Progressive accumulation of sphingolipids leads to interstitial fibrosis that becomes more prevalent and worsens in severity over time. Smooth muscle cell hypertrophy and proliferation induce intramyocardial arterioles thickening, leading to arterial stiffening. Finally, the accumulation of Gb3 causes biochemical and functional impairment of myocytes [14, 23, 24].

The typical neurological involvement in FD occurs in the small fiber, leading to peripheral neuropathy characterized by pain, cold, and heat intolerance, and in the central nervous system leading to early cerebrovascular disease. Indeed, accumulation of glycosphingolipids and thickening of the vessel walls results in the narrowing of cerebral micro-vessels predisposing to transient ischemic attack (TIA) or stroke. Of course, many other cerebrovascular accidents and TIA events are related to cardiac arrhythmias, such as atrial fibrillation or other cardiac conduction abnormalities [25]. The autonomic nervous system is also involved, and FD patients often report gastrointestinal symptoms and hyperhidrosis. Patients also experience burning pain, especially at the extremities, and pain crises that manifest early in life (2 or 3 years of age) [26], and a lower skin innervation where G3b deposition occurs has been documented in skin biopsies of patients with classic and non-classic FD [27, 28].

Although neuropathic disorders are amongst the most significant symptoms of neurological involvement in FD, symptoms of autonomic dysfunction are also present, even if they may not be as noticeable as in other neuropathic disorders. Recent research [29] has shown that autonomic neuropathy in FD presents with a spectrum of symptoms, including gastrointestinal issues, orthostatic intolerance, and sexual dysfunction. However, these manifestations are less common and more severe than expected [30–32].

Data obtained from 342 Fabry patients enrolled in the Fabry Outcome Survey (FOS) revealed that 60.8% of children and 49.8% of adults experienced gastrointestinal complaints. The most frequently reported gastrointestinal symptoms were abdominal pain and diarrhea [33].

These complaints have frequently been explained by autonomic failure: as multiple studies have shown, minor fiber damage[34] and accumulation of lipids in the autonomic ganglia in Fabry patients create symptoms and signs compatible with autonomic dysfunction [35].

Ocular manifestations are also due to glycosphingolipid accumulation in eye tissues and can be observed early in life. The main ocular signs in FD include cornea verticillate (specifically corneal epithelial opacities), conjunctival or retinal vessel alterations, and posterior cataracts [36].

Interestingly, cornea verticillate appears to be associated with a more severe disease course and with null or missense mutation rather than mild or the p.N512S mutations [37].

Dermatological signs are persistent in FD. It is estimated that over 70% of the patients show skin manifestations. The most typical signs are angiokeratomas in the pelvic region, inside the navel, and in males in the scrotum [38].

## Prognosis of fabry

Fabry Disease (FD) presents a complex clinical picture with diverse manifestations affecting multiple organ systems. The prognosis is intricately linked to the extent of organ involvement and the timeliness of diagnosis. Renal and cardiac complications notably influence life expectancy, while neurological and dermatological symptoms predominantly impact the quality of life. The array of clinical signs, coupled with non-specific symptoms and FD's rarity, frequently leads to delayed diagnosis, which can extend up to 11 years from symptom onset to definitive diagnosis, hindering timely therapeutic intervention and exacerbating organ damage [33].

Before enzyme replacement therapy (ERT), life expectancy was about 25 and 10 years shorter in males and females with FD than peers in the unaffected population. Many FD patients died before the age of 50 years, and no one lived more than 60 years. Before the introduction of renal replacement therapies (both dialysis and kidney transplantation), patients died primarily because of uremia, heart failure, arrhythmia, and syncope [39–41]. About 40% of deaths are caused by cardiovascular events, while < 10% are caused by renal and cerebrovascular events [42].

Three different phases characterize renal involvement in untreated FD male patients. The first occurs during childhood and adolescence and is characterized by glomerular hyperfiltration, usually without any alteration at urinalysis. In a subsequent phase, proteinuria, lipiduria, crystals, and Malta crosses appear in urine sediment, and the urinary concentrating or diluting capacity is altered. The last phase is characterized by severe vascular and renal involvement with progressive renal function decline and end-stage kidney disease (ESKD) development that, in males affected by the classic FD form, commonly manifests between the third and fifth decades of life [43].

Studies have shown that the early start of nephroprotective treatments and specific therapies after the initial kidney involvement prevents or reduces renal function decline. Since proteinuria is one of the most critical factors in the progression of kidney damage, its reduction helps preserve kidney function. Warnock et al. published a study demonstrating how treatment with ACE inhibitors and angiotensin receptor blockers (ARBs), In addition to ERT, increases the rate of proteinuria reduction. Moreover, patients who achieved the UPCR goal of < 0.5 mg/g had a significant eGFR slope reduction, specifically − 3.6 (− 4.8 to − 1.1) versus − 7.0 (− 9.0 to − 5.6) ml/min/1.73 m^2^/year, respectively (*p* = 0.018) [44].

Cerebrovascular manifestations are quite frequent events in FD, with an incidence of 11–25% of male patients compared to 15–21% of female patients. Typically, these events occur earlier in males (around 39.2 years old) than in females (around 51.4 years old). Similar to cardiac and renal involvement, transient ischemic attack and cerebral strokes play a significant role in the morbidity and mortality of FD [38].

In terms of survival, as reported by Waldeck et al. in a study published in 2009, which analyzed data from the Fabry Registry, males affected by FD die at a median age of 54.3 years (range 31.2–84.8), while females die at 62 years (range 39.9–76.1). The data for this analysis covers the period between 2001 (the year of approval of ERT) and 2008. Notably, deceased patients received the diagnosis of FD at an older age than living patients. Specifically, deceased males had a median age of 39.8 years, whereas living males were diagnosed at 24.4 years, and women at 55.4 years compared with 32.7 years. Furthermore, among deceased patients, not everyone had received ERT; only 81.3% of males and 41.7% of females had, with a median treatment length of 12 months and four months, respectively [42].

A more recent evaluation of the Fabry Outcome Survey data showed an essential difference between the median survival of male patients treated with ERT for five years (77.5 years) and untreated subjects(60 years) [38].

A clear genotype–phenotype correlation in FD was not established because of the elevated number of private pathogenic variants among the affected family members. At the same time, because of the heterogeneous expression of mutated genes that lead to phenotypic differences among patients, some mutations are known to be associated with both classic and attenuated disease [45]. Variants associated with the classical phenotype can include splicing defects, missense, and nonsense variants. Similarly, variants with similar characteristics can be found in patients with attenuated disease [45]. Additionally, some frequent alterations are associated with specific phenotypes and older age of symptom onset: for example the IVS4 919G > A mutation is mainly associated with concentric left ventricular inclusion hypertrophy (LVH), valvular involvement, and arrhythmias between ages 50 and 80, with limited extracardiac involvement, the N215S mutation correlates with an onset of symptoms after the age of 50, lower decrease in enzyme activity, and only myocardium impairment [46, 47].

However, with rare exceptions, predicting the phenotype and prognosis based solely on the diagnosed genetic variant is challenging. Therefore, patients should undergo evaluation and follow-up by a multidisciplinary team of expert physicians.

## FD treatment and clinical monitoring

Since 2000–2001, the natural history of FD has changed, and the prognosis has improved thanks to the introduction of targeted therapies that aim to restore impaired α-GalA enzyme activity. Currently, two classes of therapies are available for clinical use: enzyme replacement therapy (ERT) and chaperone therapy.

ERT is based on the intravenous administration of exogenous alpha-galactosidase A to replace the enzyme deficiency. The first two commercially available formulations of recombinant human α-GalA enzyme are Replagal (agalsidase alpha, iv 0.2 mg/kg/every other week) and Fabrazyme (agalsidase beta, iv 1 mg/kg/every other week). Both compounds have been approved for clinical use in different countries since the early 2000s [48]. An extended clinical experience and much evidence support their safety and efficacy [5]. Both preparations have been demonstrated to decrease plasma Gb3 and lyso-Gb3 levels, urinary Gb3 levels, Gb3 deposition in the kidney, stabilize/decrease the reduction of eGFR, stabilize/decrease left ventricular mass, to improve nerve sensitivity, gastrointestinal symptoms, pain and quality of life [5]. Both are safe and effective in improving symptoms and disease progression; no difference was found in their clinical efficacy [49]. Although they share almost the same amino acid composition, the two preparations differ in production technology, as agalsidase alpha is produced from genetically engineered human cell lines (fibroblast, human fibrosarcoma cells HT-1080). In contrast, agalsidase beta is produced in Chinese hamster ovary (CHO) cells [50].

The current recommendations highlight the importance of an early initiation of ERT treatment, but there are some minor differences between the American and European guidelines. While the European Fabry Disease Network (EFDN) suggests that for classically affected males, ERT is recommended as soon as there are early clinical signs of kidney, heart, or brain involvement and may be considered in patients of ≥ 16 years even in the absence of clinical signs or symptoms of organ involvement [51], the American College of Medical Genetics and Genomics (ACMG) guidelines recommend that ERT be initiated as early as possible in all males with Fabry disease (including children and those with ESKD undergoing dialysis and kidney transplantation), as all are at high risk for renal, cardiac, and cerebrovascular complications [52].

In males with classic FD, ERT should be initiated in both symptomatic and asymptomatic individuals, it is appropriate at any age of presentation and should be considered also before adulthood (< 18 years old) [4]. Indeed, it has been shown that men with classical FD who started ERT before age 25 had a more significant reduction of plasma lyso-Gb3 and a lower risk of developing antibodies [53]. In late-onset males and classic/late-onset females, ERT should also be considered in asymptomatic individuals with evidence of central organ involvement (this includes GFR < 90 mL/min/1.73 m^2^, persistent albuminuria > 30 mg/g, podocyte foot process effacement or glomerulosclerosis on renal biopsy, moderate or severe Gb3 inclusions in renal cell types; silent strokes, cerebral white matter lesions on brain MRI; asymptomatic cardiac disease such as cardiomyopathy or arrhythmia, cardiac fibrosis on contrast cardiac MRI) [4, 51].

Despite their efficacy, ERTs present several limitations. ERT may not fully reverse FD physiopathology and clinical manifestations of FD. ERT has limited bioavailability due to poor permeability through the blood–brain barrier. Additionally, adverse reactions to infusions have been reported, and ERT requires long-term intravenous administration, potentially leading to antibody responses.

Studies showed that anti-drug antibodies (ADAs) usually develop in three to six months after the ERT start, are most frequent in males affected by classic FD, and a cross-reaction was demonstrated with agalsidase alfa and beta [54–56]. Inhibitory anti-drug antibodies are mainly IgG1 and IgG4 [57]. They may affect ERT efficacy by binding to the enzyme, leading to macrophage activation, antibody-ERT complexes internalization, and enzyme inactivation [55]. The reduced target cellular uptake of the enzyme reduces the effectiveness of ERT and is associated with worse renal and cardiac outcomes and worsening of the patient’s symptoms [58]. Hence, it is of primary importance to know the serostatus of patients through the identification of ADAs, as demonstrated by Lenders et al. [58] by serum-mediated inhibition assays followed by titration assays to determine the individual inhibitory capacities of ADAs.

To mitigate the adverse effects of anti-drug antibodies, a potential strategy involves increasing the dose of ERT. A recent study demonstrated that dose escalation has varied long-term effects on patients. It appears to cause an increase in ADA titers after the switch, but it could also result in sustained ADA saturation. Regardless of an increase in ADA titers, lyso-Gb3 levels decrease, and cardiac and renal parameters remain stable over time in patients who experienced a fivefold dosage increase of ERT [59]. A different approach was evaluated to prevent ADA formation by administering immunosuppressive therapy, as shown in an analysis of Fabry patients who received immunosuppressive drugs for kidney or heart transplantation. This demonstrated its efficacy in preventing de novo ERT inhibition in ERT naive patients [60]. However, while higher doses of immunosuppressive medications are linked with reduced antibody levels and less ERT inhibition in affected patients, they do not provide lasting protection, and these drugs' side effects must be considered.

In 2016, Galafold (migalastat, *per os* 123 mg/every other day), the first-in-class pharmacological chaperone therapy for FD, was approved in Europe. It is an iminosugar analog of the terminal galactose residue of Gb3 and acts as a competitive inhibitor of the α-GalA enzyme. Migalastat selectively and reversibly binds to the active site of alpha-galactosidase A. This binding stabilizes the α-GalA enzyme, facilitating its proper trafficking from the endoplasmic reticulum into the lysosome. Once in the lysosome, the lower pH and the higher concentration of substrates lead to the dissociation of migalastat from the endogenous enzyme, allowing it to exert its action (Gb3 and related substrates catabolism). Its efficacy has been demonstrated in the FACETS trial, where migalastat was compared to placebo, and in the ATTRACT trial, where it was compared to ERT. In both studies, treatment with migalastat effectively reduced the left ventricular mass index and stabilized the eGFR [61, 62].

Because of its characteristics, migalastat shows different advantages compared to ERT. It is administered orally, has a better tissue distribution (small molecule), induces sustained and stable enzyme levels, and does not exhibit immunogenicity [5]. On the other hand, chaperone therapy is a therapeutic option only for patients with ‘amenable’ variants that result in abnormally folded or less stable proteins with retained enzymatic activity (a list of GLA amenable variants is reported in the Galafold product information document released by EMA and FDA and is accessible online at https://www.galafoldamenabilitytable.com). The amenability of GLA variants is evaluated through an in vitro assay, in which Human Embryonic Kidney (HEK-293) cell lines are transfected with specific variants and produce mutant proteins. A variant is amenable when the enzyme activity shows an increase of at least 20% compared to the pre-treatment and an absolute increase of at least 3% of the wild-type α-GalA activity. Thus, chaperone therapy cannot be used for treatment in patients with a mutation causing a severe enzyme activity depletion (i.e., gene deletion leading to no endogenous α-GalA enzyme activity). Of note, in vitro amenability does not reflect the effectiveness of the chaperone treatment, as the in vitro assay may not evaluate mutant proteins trafficking into the lysosome or the dissociation of migalastat from the mutant proteins within the lysosome [5, 63].

Watching the behavior of individuals with Fabry Disease is essential for efficiently managing their clinical care. Despite putative biomarkers associated with biological activity in Fabry disease have been researched, the ideal biomarker for clinical application has yet to be identified. As a result, other instruments already validated to assess and follow disease progression are necessary. The initial two scoring systems designed to measure the severity of Fabry disease were the Mainz Severity Score Index (MSSI) and the Fabry Disease Severity Scoring System (DS3) [2, 64]. Both of these systems necessitate the evaluation of multiple domains, with a considerable number of items to consider (DS3 includes five domains and 12 items, while MSSI consists of 4 domains and 24 items). As a result, calculating and implementing these indices can be time-consuming and potentially challenging. Additionally, certain items within these indices heavily rely on symptom ratings reported by the patients themselves. Lastly, these tools only provide a snapshot of disease severity at a specific moment, failing to estimate the clinical variation compared to previous visits. In 2016, a group of Italian experts developed a dynamic mathematical model [the FASTEX (Fabry Stabilization indEX)] to assess clinical stability. First is a simplified scoring system called the Raw Score (RW). This score evaluated seven parameters that are easy to access: neuropathic pain, cerebrovascular events, albuminuria and proteinuria, estimated glomerular filtration rate (eGFR), echocardiographic and electrocardiographic alterations, as well as the New York Heart Association (NYHA) classification. Each of these parameters is graded from 0 to 4 based on the severity of the clinical manifestation. Subsequently, the Weighted Score (WS) has been introduced to represent the true impact of each clinical manifestation as a percentage. After extensive research, a mathematical algorithm was created to calculate the extent of deterioration in patients' conditions. This algorithm involves entering the RS score from a previous visit and the RS score from the second most recent visit to obtain a percentage value known as the FASTEX score. It was unanimously agreed that a FASTEX score of 20% would serve as a threshold for disease stability [1].

## Future perspectives of personalized medicine in FD

Research into new treatments for FD has intensified due to the limitations of currently available therapies. Investigations are underway for novel approaches like new enzyme replacement therapy (ERT), substrate reduction therapy (SRT), mRNA therapy, and genetic therapy [5].

Two intriguing molecules have been studied to extend ERT possibilities. Pegunigalsidase alfa is a modified alfa galactosidase A produced from a plant-based cells system with a PEGylation, which determines a higher stability and half-life and a different biodistribution profile from agalsidase alfa and beta, which could allow monthly infusion as is under investigation in an open-label study to assess efficacy and safety of this distinct dose regimen (NCT03614234). Pegunigalsidase alfa was approved by the FDA in 2023 since it has been proven to be effective in reducing plasma lyso-Gb3 levels and its deposition in the kidney, slowing eGFR slope and proteinuria levels, as well as deposition in the left ventricle. Additionally, PEGylation appears to mask some epitopes from the immune system, reducing immunogenicity. Studies have even shown that pegunigalsidase can reverse anti-drug antibodies and induce immune tolerance [65–67], and it has been proven to be non-inferior to the two previously approved ERTs [68, 69]. A second ERT molecule under development is the moss-derived alfa-galactosidase A. This enzyme is derived from *Phycomitrella patens*. It has been designed to elude the immunogenic response. Although it has a shorter half-life due to increased cellular uptake via the mannose receptor, it has been demonstrated that it induces a reduction of urinary Gb3 [70, 71].

Another approach to reducing globotriaosylceramide deposits interferes with its synthesis by blocking the glucosylceramide synthetase (GCS), the enzyme responsible for the initial biosynthesis step (Substrate Reduction Therapy—SRT). Two drugs currently under investigation for this purpose are ibiglustat (Venglustat) and lucerastat. These drugs aim to reduce Gb3 to a level that can be catabolized by the residual activity of α-GalA (non-classical mutations). Alternatively, SRT can be added to ERT if α-GalA activity is lower than 1%. The oral administration and the penetration to the blood–brain barrier are the main advantages of SRT [5, 72] however, these compounds are still not available for routine prescription, and clinical trials are ongoing to evaluate their efficacy and safety (NCT0528054, NCT05206773, NCT03737214) Recently, a new glucosylceramide synthetase inhibitor (AL01211) has been assessed in a phase-1 trial in which safety, pharmacokinetics, and pharmacodynamics effects were studied. It is currently under investigation in naive male subjects affected by the classic disease (NCT06114329).

RNA therapy involves mRNA molecules encapsulated in lipid nanoparticles, which deliver GLA to hepatocytes. This allows hepatocytes to produce the correct protein and secrete it into circulation. Studies in mice and non-human primates demonstrated an increase in enzyme activity in a dose-dependent manner, along with reductions in Gb3 and lyso-Gb3 levels [73, 74].

A functional GLA gene is introduced into cells using either viral or non-viral vectors in gene therapy. Generally, these therapies rely on targeted cells overexpressing the interested gene, while other cell lines take up GLA via mannose-6-phosphate receptors, transporting it to the lysosomes. However, the lack of specific vectors for the heart and kidney poses challenges for gene therapy. Currently, the gene is primarily transported to hepatic cells or HSPCs (hematopoietic stem and progenitor cells), where it is transcribed and translated into enzymes, released in circulation, and taken up by other cell tissues [75–77].

In addition to specific therapies, cardio- and nephro-protection are fundamental to slow disease progression, especially when organ damage is already established. With this aim, renin–angiotensin–aldosterone system inhibitors (RAASi) have been used as an add-on ERT therapy with a further positive effect on renal function protection [78].

Over the last years, the inhibitors of sodium-glucose co-transporter 2 (SGLT2i) have been massively studied for their antiproteinuric, natriuretic nephro- and cardio-protective effects [79]. At the moment, there is one clinical trial that aims to evaluate the effect of SGLT2i (Dapagliflozin) on albuminuria, kidney disease progression and cardiologic involvement and exercise capacity and quality of life in patients with FD and CKD stages 1 to 3 [80].

Braun et al. published a study in which alfa-synuclein's possible role in podocyte injury was established. Using a proteomic and transcriptomic approach, the authors have identified an upregulation of alfa-synuclein in GLA-deficient podocytes [81]. This could be an additional pathogenic mechanism explaining why even though ERT significantly reduces the volume of Gb3 inclusions in the kidney, podocyte injury does not improve. Moreover, this could be a possible future therapeutic target, even if more studies are needed.

Currently under research but hopefully usable soon is the possibility to study transcriptional data of nephron compartments (glomeruli, tubules, interstitium, arteries) from renal biopsies. This can help to identify a priori the response to enzyme replacement therapy or the possible expression of ERT-resistance genes. Studying transcriptional products can also help evaluate when ERT should be started because, as known, mRNA expression precedes clinical alterations [62].

## Conclusion

Undoubtedly, Fabry Disease (FD) is now on the cusp of a transformative era characterized by personalized medicine. Soon, diagnosis, therapies, and follow-up will be customized based on individual and specific factors such as gene mutation types and gene expressions and the severity of organ involvement. While effective to some degree, the one-size-fits-all approach will likely give way to a combination of drugs tailored to the residual α-GalA residual activity or guided by the presence and titer of ERT antibodies. Additionally, management will also be guided by the specific gene mutation types.

Current and emerging supportive therapies will undoubtedly enrich this individualized treatment paradigm. By synergizing specific, person-centered therapies with cardio-renal protective strategies, we will likely witness a significant enhancement in the survival rates and overall quality of life for those afflicted by FD. This shift from a standard to a precision-based treatment model reflects the field's progress and underscores a future where medical interventions can be as unique as the individuals they aim to heal.

## Abbreviations

CKD: Chronic kidney disease
eGFR: Estimated glomerular filtration rate
EMT: Epithelial-mesenchymal transition
ERT: Enzyme replacement therapy
ESKD: End-stage kidney disease
FD: Fabry disease
Gb3: Globotriaosylceramide
GCS: Glucosylceramide synthetase
GFR: Glomerular filtration rate
GLA: α-Galactosidase A gene
α-GalA: α-Galactosidase A (enzyme)
lyso-Gb3: Globotriaosil-sphingosine
SRT: Substrate reduction therapy
SRT: Substrate Reduction Therapy
